# Absence of Ribosome Modulation Factor Alters Growth and Competitive Fitness of Escherichia coli

**DOI:** 10.1128/spectrum.02239-21

**Published:** 2022-04-04

**Authors:** Hans Sebastian, Steven E. Finkel

**Affiliations:** a Molecular and Computational Biology Section, Department of Biological Sciences, University of Southern California, Los Angeles, California, USA; Northwestern University

**Keywords:** 100S ribosome, competitive fitness, long-term survival, ribosome modulating factor

## Abstract

During stationary phase in Escherichia coli, the expression of the ribosome modulation factor (RMF) protein participates in the dimerization of two 70S ribosomes, ultimately creating a 100S particle. 100S ribosomes are commonly thought to function to preserve ribosomes as growth ceases and cells begin to catabolize intracellular components, including proteins, during their transition into stationary phase. Here, we show that the rates of stationary-phase ribosomal degradation are increased in an *rmf* mutant strain that cannot produce 100S ribosomes, resulting in deficiencies in outgrowth upon reinoculation into fresh medium. Upon coinoculation in LB medium, the mutant exhibits a delay in entry into log phase, differences in growth rates, and an overall reduction in relative fitness during competition. Unexpectedly, the *rmf* mutant exhibited shorter generation times than wild-type cells during log phase, both in monoculture and during competition. These doubling times of ∼13 min suggest that failure to maintain ribosomal balance affects the control of cell division. Though the timing of entry into and exit from log phase is altered, 100S ribosomes are not essential for long-term viability of the *rmf* mutant when grown in monoculture.

**IMPORTANCE** Ribosomes are the sole source in any cell for new protein synthesis that is vital to maintain life. While ribosomes are frequently consumed as sources of nutrients under low-nutrient conditions, some ribosomes appear to be preserved for later use. The failure to maintain the availability of these ribosomes can lead to a dire consequence upon the influx of new nutrients, as cells are unable to efficiently replenish their metabolic machinery. It is important to study the repercussions, consequences, and mechanisms of survival in cells that cannot properly maintain the availability of their ribosomes in order to better understand their mechanisms of survival during competition under nutrient-depleted conditions.

## INTRODUCTION

Ribosomes are a vital component of the cell due to their unique role in synthesizing the proteins needed for proper cell growth, metabolism, and stress response. Disturbances in the activity of this complex macromolecule can impair a cell’s ability to properly respond to changes in the environment. In Escherichia coli, one protein that is central to the regulation of ribosomal activity is the ribosome modulation factor (RMF) (1–6). RMF primarily functions as the initiator, along with the hibernation promoting factor (HPF), in the two-step process of binding two 70S ribosomes to form a 100S ribosome dimer (2). The transcription of the *rmf* gene initiates with the accumulation of guanosine tetraphosphate (ppGpp) that occurs when reduced levels of substrates for protein synthesis are detected in the cell (1). *rmf* transcription can also be triggered by the binding of cyclic AMP receptor protein to the *rmf* promoter as the cell detects a decrease in metabolic energy (7). The translation of the *rmf* gene is enhanced by the presence of polyamines (8).

In addition to participating in the creation of ribosome dimers, RMF in E. coli has been proposed to modulate several cellular stress responses. These responses include heat resistance (9), rRNA protection under acidic conditions (10), and prevention of ribosomal degradation (3, 11). These responses, however, and their direct relationship to the formation of 100S ribosomes, remain to be fully characterized. Due to the various cellular processes that RMF affects, the loss of this protein is known to negatively impact fitness (4). However, some evidence challenges this common assumption, and little is known about RMF activity during long-term stationary phase (LTSP) (12), calling for further study of the role of RMF in long-term fitness of the cell.

E. coli cells grown in batch culture display five distinct phases when incubated in a rich medium, such as Luria-Bertani (LB) broth (13). The first, lag phase, is characterized as the period when cells sense available nutrients upon inoculation into a new environment and retool their metabolism to enter exponential- or logarithmic-phase growth (14). Upon entry into exponential phase, cells divide rapidly, with a typical generation time of ∼20 min, routinely reaching cell densities of ∼5 × 10^9^ CFU/mL (15). Subsequently, as cells enter stationary phase, division halts as readily metabolizable nutrients in the medium decrease and metabolic by-products accumulate. During stationary phase, many of E. coli’s stress responses are activated (16). These responses include the following: the conversion of the cellular morphology to smaller, more coccoid cells, with a relative increase in periplasmic volume, to increase envelope resistance (17); the expression of the Dps protein, which condenses the nucleoid into the biocrystal nucleoprotein complex (18); the starvation stress response upon the accumulation of the stationary-phase-specific sigma factor RpoS, which regulates the transcription of ∼23% of the genome (19, 20); and the production and accumulation of the alarmone ppGpp, which induces the production of RMF (1). Although the length of the stationary phase is strain and medium specific (21), the population will eventually enter the death phase, when ∼99% of cells lose viability. Ultimately, the surviving population enters the fifth phase, a period of dynamic equilibrium of cell growth and death known as LTSP (13).

Through the first two phases, RMF levels remain relatively low until cells transition into stationary phase. During this time, 100S ribosomes form and have been reported to be responsible for modulating the rate of ribosomal degradation that occurs during the entry into stationary phase (22–25). This ribosomal degradation in stationary phase can be visualized by observing cells stained with acridine orange (26). Most cells during log phase appear to be replete with rRNA (staining bright orange), whereas cells from 3-day-old stationary-phase cultures show a significant reduction in rRNA levels (staining green) (Debby Siegele and Robert Kolter, personal communication). While ribosome degradation is known to occur as cells enter stationary phase (27), Fukuchi et al. (28), Wada (3), and others (9, 29) showed increased degradation of ribosomes in *rmf* mutants of E. coli when stressed or entering a prolonged period of stationary phase. Another Gram-negative bacterium, Pseudomonas aeruginosa, also exhibited increased ribosomal degradation when HPF, a protein involved in hibernation, was absent (30). This phenomenon also occurred in Gram-positive bacteria, which displayed increased ribosomal degradation during hibernation when deficient in the long form of HPF, the sole hibernation protein in these Gram-positive microbes (25, 31). In addition, Yamagishi et al. reported a loss of viability in *rmf* mutant cells after 4 days in batch culture (4). Due to the reported instability of ribosomes in stationary phase caused by the lack of hibernation proteins maintaining the ribosomes in the cell, we hypothesized that an *rmf* mutant of E. coli would exhibit a defective outgrowth phenotype through the lag and log phases upon reinoculation from a stationary-phase culture into fresh medium, since the lack of ribosomes would cause a delay in the ability of cells to rapidly synthesize proteins required for exponential-phase growth. Furthermore, we proposed that the mutant would suffer a significant loss of viability during the long-term stationary phase due to increased rates of ribosomal degradation, as *rmf* mutant cells lose the ability to protect ribosomes that is conferred by RMF-mediated 100S particle formation (3).

Here, we show that the absence of RMF impacts the timing of the transition from lag phase into log phase, resulting in reduced competitive fitness. Furthermore, we observe differences in the amounts of rRNA in wild-type cells compared to the amounts in *rmf* mutant cells. The observed phenotypes support a model where *rmf* mutant cells experience greater amounts of ribosomal degradation during stationary phase, extending the length of the lag phase upon inoculation into fresh medium. Our data suggest that the ability to stabilize and preserve ribosomes within a 100S particle enables cells to recover more quickly following nutrient stress, as they do not have to expend as much time and energy during lag phase to regenerate their ribosome pool.

## RESULTS

### *rmf* mutant growth is impaired in coculture but unaffected in monoculture.

**FIG 1:**
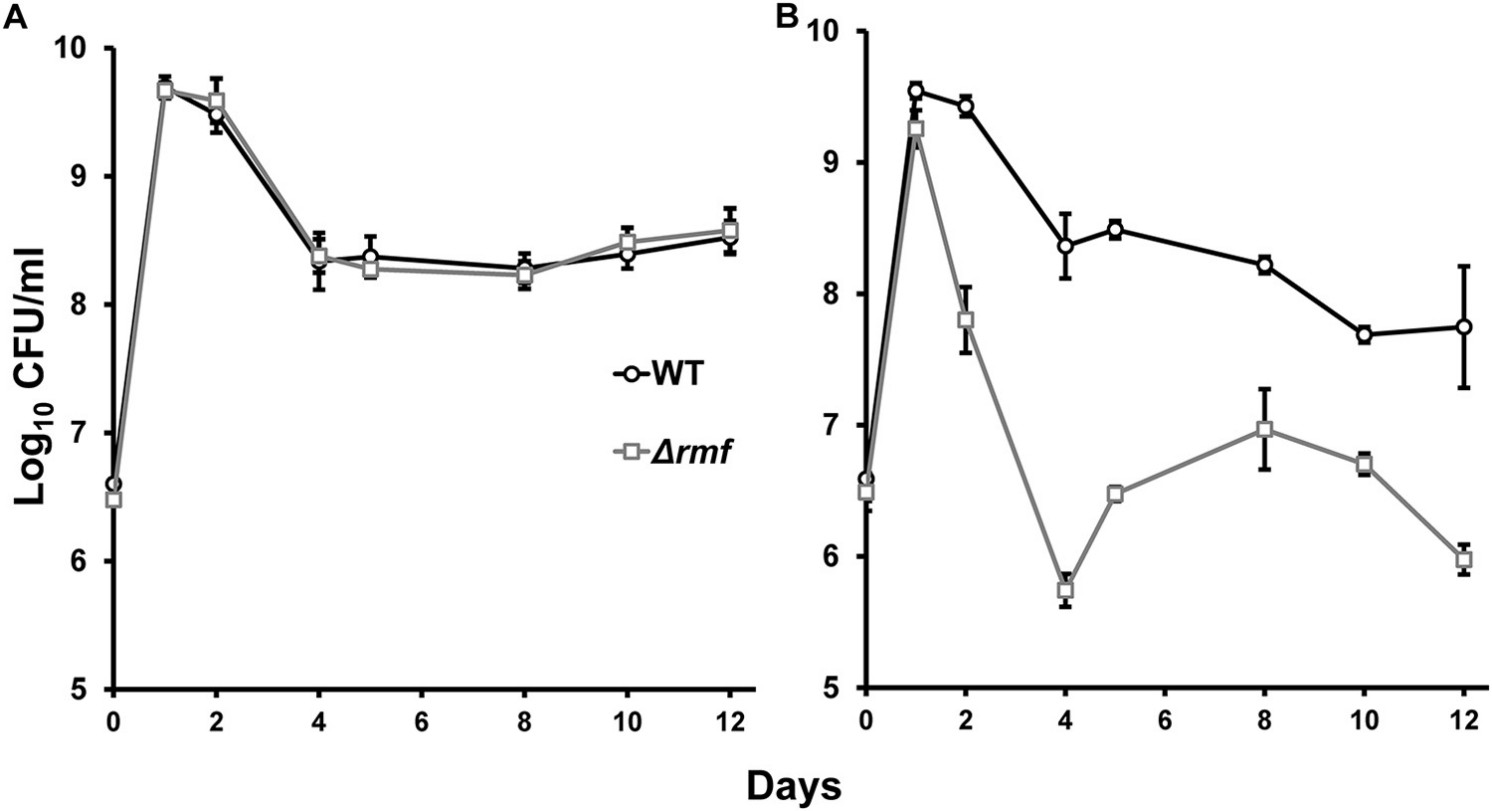
Growth of the *rmf* mutant is impaired in the presence of wild-type cells. Monoculture (A) and coculture (B) growth of wild type (open circles, black lines) and mutant (open squares, gray lines) is shown. Lines represent average viable cell counts (CFU/mL). Error bars represent the standard deviations; *n* = 3.

To examine the consequences of a mutation in *rmf* for the long-term survivability of E. coli, wild-type and *rmf* mutant strains were incubated in monoculture or coculture to determine cell yields and relative fitness in both types of culture environment. Despite previous reports of impaired survival (4), we observed that *rmf* mutant cells displayed the same ability as wild-type cells to survive in monoculture, despite their inability (4) to form 100S ribosomes during stationary phase (Fig. 1A). Strains inoculated independently at a cell density of ∼5 × 10^6^ CFU/mL both reached a maximum cell yield of ∼5 × 10^9^ CFU/mL by day 1 and maintained this density for 2 days in stationary phase before entering the death phase. Through day 12, both strains maintained similar viable cell counts through the long-term stationary phase at cell densities of ∼5 × 10^8^ CFU/mL. This was in stark contrast to the many differences in the growth and survival patterns observed when the *rmf* mutant was cocultured with wild-type cells (Fig. 1A and B). First, the overnight (day 1) cell yield of the *rmf* mutant was consistently 10% to 50% of the wild-type strain yield. Second, the *rmf* mutant suffered a more severe death phase than the wild-type cells, with cell densities at least 40-fold lower than the densities of the wild-type cells on day 2 and a further decay of more than 1,000-fold compared to the wild-type by day 4. Third, while the *rmf* mutant showed a more severe decrease in the viable cell count during death phase, the *rmf* mutant began to partially recover from this “dip” and regrow about 10-fold as cells transitioned into the LTSP. Fourth, despite the brief growth recovery, the wild-type cell densities during long-term stationary phase remained ∼100 times greater than the cell densities of the *rmf* mutant, while the mutant cell viable counts continued to decrease. These four phenomena were not observed in the monoculture growth of the *rmf* mutant (Fig. 1).

### *rmf* mutant cells display impaired growth in early log phase in both monoculture and coculture.

There are several mechanisms, not mutually exclusive, that may explain the observed differences in overnight cell yields between the *rmf* mutant and wild-type cells during coculture competitions. One possibility is that the *rmf* mutant has a lower growth rate, resulting in a lower cell density following log phase. Another possibility could be that the *rmf* mutant and wild-type cells exhibit the same log-phase growth rate but differ in the timing of their transition into log phase. This would cause the mutant to enter log phase later than the wild type and reach a lower final cell density.

**FIG 2:**
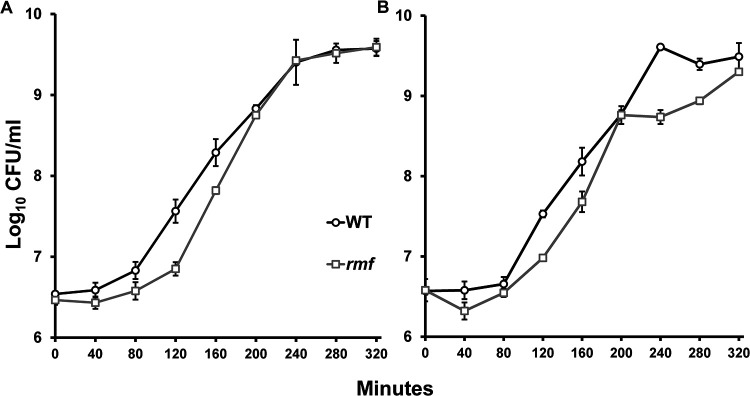
Altered log phase exhibited by the *rmf* mutant in both monoculture and coculture. Monoculture (A) and coculture (B) growth of wild type (open circles, black lines) and mutant (open squares, gray lines) during the first 320 min of outgrowth is shown. Lines represent average viable cell counts (CFU/mL). Error bars represent the standard deviations; *n* = 3.

To distinguish between these hypotheses, we performed growth rate assays under both monoculture and coculture conditions, determining the titers of cultures every 40 min to determine the lengths of the lag phase and the exponential growth rates. The duration of lag phase was determined by viable cell count, rather than by spectrophotometry, because optical density measurements are not sensitive enough to accurately monitor the changes in cell numbers associated with the transition from lag phase to early log phase (32). When grown in monoculture (Fig. 2A), the cell counts for both wild-type and mutant strains were similar for the first 80 min. However, after 120 min, only the wild-type cells entered log phase, whereas the mutant-cell density was an order of magnitude lower. Interestingly, when the *rmf* mutant did enter log phase, its growth rate was extremely high, with a generation time of ∼12.8 min compared to ∼19.4 min for the wild type. To confirm this, we performed a series of paired *t* tests along the growth curve to test for significant differences in CFU/mL, and we found that the 120- and 160-min time points differed significantly between the wild type and the mutant, with *P* values of 0.004 and 0.03, respectively (Fig. 2A). The growth rates were calculated (see Materials and Methods) through fitting a linear trendline on the exponential-phase data from minutes 80 to 240 for the wild type and 120 to 200 for the *rmf* mutant and tested for significance using the *t* test (*P* = 0.012). After 200 min postinoculation, both mutant and wild-type cells reached similar densities until the end of the experiment (minute 320).

When incubated in coculture, however, the *rmf* mutant again showed distinct phenotypic differences during outgrowth (Fig. 2B). Similar to the results in monoculture, the mutant displayed a slower transition to log phase than did wild-type cells. Furthermore, the growth of the *rmf* mutant was once again very rapid by mid-log phase, with a generation time of ∼13.6 min, compared to ∼19.5 min for wild-type cells, from 120 to 200 min. Unlike that of the wild-type cells, the *rmf* mutant cell density reached a plateau for ∼40 min, while the wild-type cells maintained their exponential trajectory to reach stationary phase. Ultimately, the *rmf* mutant cells resumed growth and approached close to the wild-type cell density as they entered stationary phase. Similar to the analysis of the monocultures, the growth rate was calculated for the competition culture (see Materials and Methods) through fitting a linear trendline on the exponential-phase data and testing for significance using paired *t* tests (*P* = 0.027). In addition, we performed a series of paired *t* tests along the exponential phase to test for significant differences in CFU/mL, and we found that the 80-, 120-, 160-, and 240-min time points differed significantly between the wild type and the mutant, with *P* values of 0.018, 0.002, 0.001, and 0.001, respectively (Fig. 2B).

### The longer lag phase of the *rmf* mutant is correlated with the presence of fewer ribosomes.

**FIG 3:**
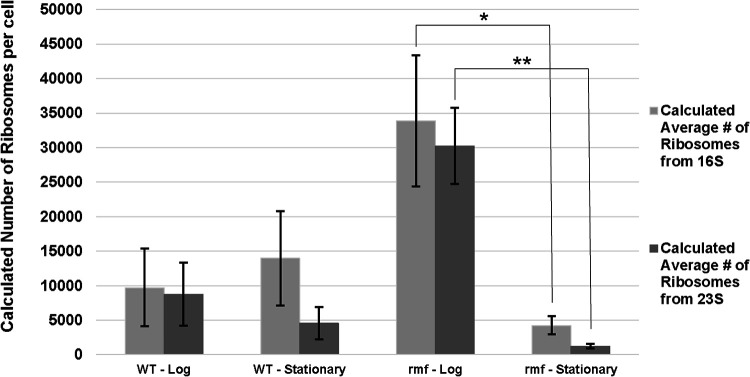
Comparison of calculated numbers of ribosomes per cell between growth phases in the wild-type and *rmf* mutant strains. Averages of the estimated numbers of ribosomes are shown, with error bars representing the standard deviations. Two-sample *t* test was performed to determine significant differences between the growth phases for each strain (*, *P* < 0.05; **, *P* < 0.01).

We used the amounts of 16S and 23S rRNA (see Materials and Methods) as a proxy to calculate how many ribosomes were present in the cell, assuming that the amount of rRNA should be directly proportional to the number of ribosomes (33, 34). At minute 150 of monoculture growth, the number of ribosomes in the wild type was 0.29 times that of the mutant cells (Table 1, Fig. 3). However, during stationary phase, the situation was reversed, and the number of ribosomes present in wild-type cells was ∼3.5 times greater than the number in the *rmf* mutant (Table 1, Fig. 3).

**TABLE 1:** *rmf* mutant has more ribosomes than the wild type during log phase but fewer during stationary phase^*a*^

Phase	Sample	Area percentage (*f*) of:		RNA concn (pg/μL)	Total RNA (pg) (*C*)	CFU (*d*)	Total amt (pg) of:		No. of ribosomes from:		Avg no. of ribosomes (*R*) from:		Ratio of wild type to *rmf* from:	
		16S rRNA	23S rRNA				16S rRNA	23S rRNA	16S rRNA	23S rRNA	16S rRNA	23S rRNA	16S rRNA	23S rRNA
Exponential	WT-1	20.0%	34.5	4.99E+04	4.99E+07	7.20E+08	1.39E−02	2.39E−02	1.37E+04	1.20E+04	9.71E03 ± 5.63E03	8.76E03 ± 4.59E+03	0.29	0.29
	WT-2	17.8	33.7	7.17E+04	7.17E+07	2.20E+09	5.80E−03	1.10E−02	5.73E+03	5.51E+03				
	rmf-1	15.2	29.0	5.64E+04	5.64E+07	3.12E+08	2.75E−02	5.24E−02	2.71E+04	2.63E+04	3.39E04 ± 9.51E03	3.02E04 ± 5.54E03		
	rmf-2	17.4	28.8	6.33E+04	6.33E+07	2.68E+08	4.11E−02	6.80E−02	4.06E+04	3.42E+04				
Stationary	WT-1	17.4	10.9	2.65E+04	2.65E+08	2.50E+09	1.85E−02	1.16E−02	1.82E+04	5.80E+03	1.40E04 ± 6.82E03	4.54E03 ± 2.34E03	3.30	3.75
	WT-2	17.2	10.2	9.70E+03	9.70E+07	2.70E+09	6.18E−03	3.66E−03	6.10E+03	1.84E+03				
	WT-3	17.6	11.8	1.72E+04	1.72E+08	1.70E+09	1.78E−02	1.19E−02	1.76E+04	5.99E+03				
	rmf-1	13.8	8.2	1.07E+04	1.07E+08	3.30E+09	4.47E−03	2.66E−03	4.41E+03	1.33E+03	4.23E03 ± 1.31E03	1.21E03 ± 3.35E02		
	rmf-2	12.1	7.0	6.04E+03	6.04E+07	2.55E+09	2.87E−03	1.66E−03	2.83E+03	8.33E+02				
	rmf-3	12.6	6.7	7.65E+03	7.65E+07	1.75E+09	5.51E−03	2.93E−03	5.44E+03	1.47E+03				

a Comparison of the differences in numbers of ribosomes that exist between the wild-type strain, ZK1142, and the *rmf* mutant strain, SF2603, (*n* = 3) within log-phase and stationary-phase cells.

### Normal growth pattern is restored in the *rmf* mutant when reinoculated from log-phase monocultures.

We reasoned that wild-type cells should be replete with ribosomes by the end of the lag phase, throughout the log phase, and early in the stationary phase. In contrast, *rmf* mutant cells may not have fully replenished their ribosome pools during early lag phase because of the deficit they experience due to ribosome degradation during the previous stationary phase, prior to inoculation into fresh medium. Therefore, when *rmf* mutant cells are reinoculated into fresh medium from an overnight stationary-phase culture, those cells will exhibit a growth defect. To test this, we again incubated both strains in either monoculture or coculture. However, here, rather than inoculating from overnight cultures, we started a fresh set of cultures and, at different time points postinoculation (120, 150, 200, and 320 min), used these cultures as the inocula for another set of cultures with fresh medium and monitored the viable cell counts every 40 min. This allowed us to compare the growth of (i) cells that might have still been in lag phase, (ii) cells that might have already entered log phase, and (iii) cells that were exiting log phase and transitioning into stationary phase.

**FIG 4:**
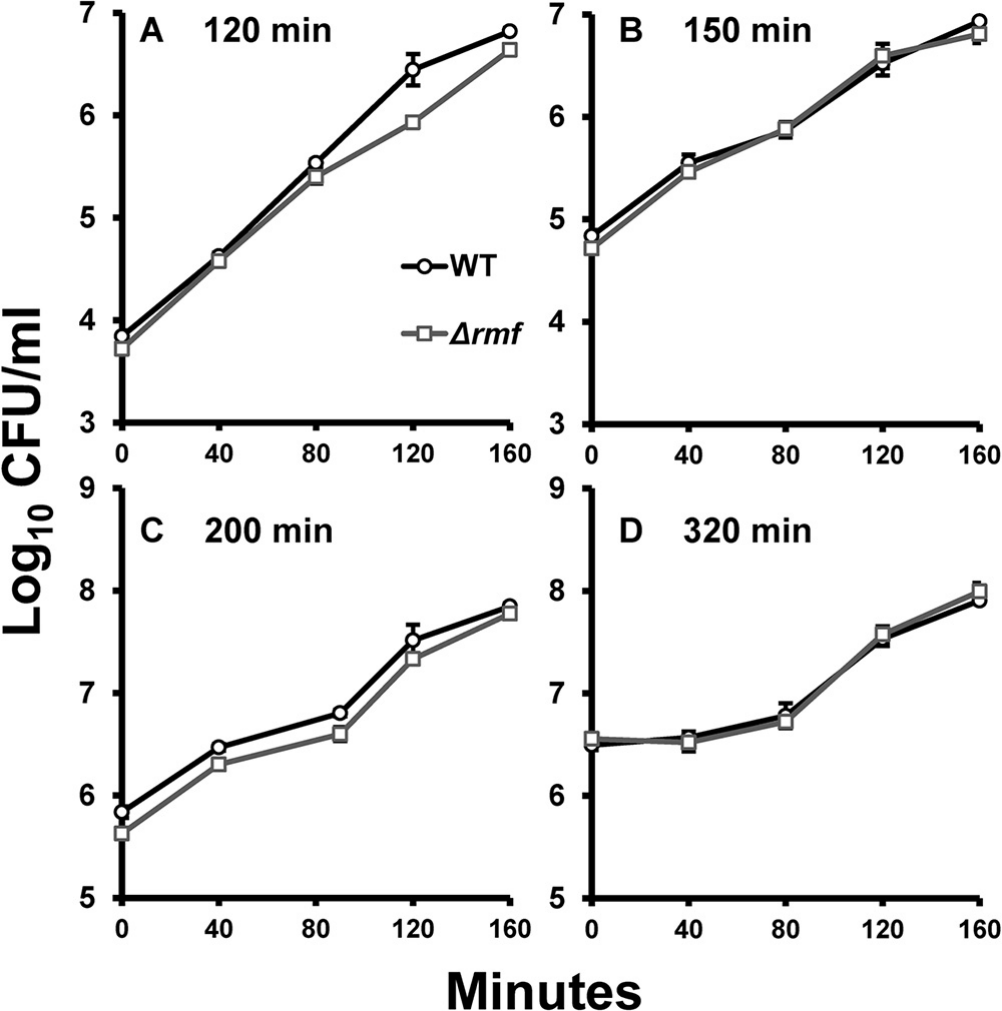
Reinoculation of mutant cells during monoculture growth restores the wild-type phenotype. From monocultures of wild type (black) and mutant (gray), 1:1,000 dilutions were made at the following times: 120 min (A), 150 min (B), 200 min (C), and 320 min (D). Thin black lines and gray lines represent wild-type growth and mutant growth, respectively. Bold lines with open circles (wild type) and open squares (*rmf* mutant) represent the average CFU/mL of the replicates; *n* = 3.

In monoculture, *rmf* mutant cells harvested from the earliest time point (minute 120) took longer to reach cell densities comparable to those of wild-type cells (Fig. 4A). Despite the growth pattern of the *rmf* mutant being similar to that of the wild type for the first 80 min, wild-type and mutant cell growth differed at minute 120 post-reinoculation (*t* test; *P* = 0.033), perhaps due to the mutant cells having not fully replenished their ribosomal pool (Fig. 4A). For reinoculations from the minute 150, 200, and 320 time points (Fig. 4B to D), the growth of the *rmf* mutant tracked with that of the wild type, suggesting that the ability of the mutant to exhibit wild-type-like growth was restored later in monoculture. All time points were tested for significant differences using the paired-sample *t* test. Significant differences were only found for the 80-, 120-, and 160-min time points of reinoculation from the minute 120 time point (Fig. 4A).

**FIG 5:**
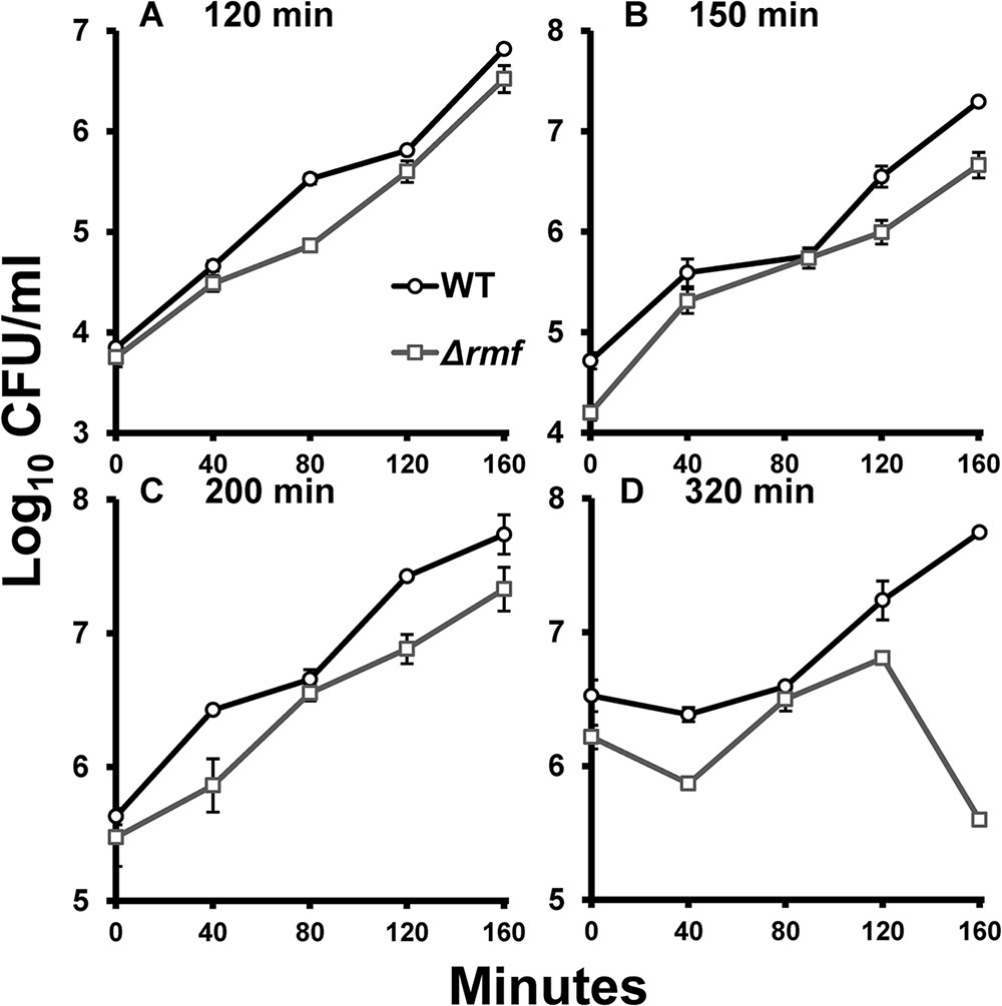
Reinoculation of coculture does not restore wild-type phenotype to the mutant. From competition cultures of wild type (black) and mutant (gray), 1:1,000 dilutions were made at the following times: 120 min (A), 150 min (B), 200 min (C), and 320 min (D). Thin black lines and gray lines represent wild-type growth and mutant growth, respectively. Bold lines with open circles (wild type) and open squares (*rmf* mutant) represent the average CFU/mL of the replicates; *n* = 3.

In contrast, when reinoculating cells harvested from competition cultures during lag or log phase, the presence of the wild type seemed to impair the growth of the *rmf* mutant (Fig. 5A to D) at any time point of reinoculation. At the earliest time point of reinoculation (minute 120), the mutant experienced slow growth, suggesting that the *rmf* mutant was less fit than the wild-type cells (Fig. 5A). This observation was not unexpected, since the mutant showed a similar phenotype in monoculture (Fig. 4A), even though it was able to return to a similar cell density at minute 160 post-reinoculation. However, at later times of harvest and reinoculation, the mutant’s growth continued to be impaired in the presence of wild-type cells (Fig. 5B to D). Furthermore, an additional competition-specific phenotype was observed in cultures reinoculated from minute 320 starter cultures (Fig. 5D). Here, the *rmf* mutant showed a significant loss of viability, with final cell yields about 100-fold lower than those of the parental strain, whose growth was unaffected. To confirm significance, paired-sample *t* tests were applied to the growth curve data. Significant differences were observed for the following time points: minute 80 (Fig. 5A) (*P* = 0.003), minutes 40 and 120 (Fig. 5C) (*P* = 0.036 and 0.012, respectively), and minutes 0, 40, 120, and 160 (Fig. 5D) (*P* = 0.005, 0.004, 0.025, and 0.00002, respectively).

## DISCUSSION

Ribosome hibernation and inactivation are phenomena that occur in E. coli as a response to stress and low-nutrient conditions (1–6). Several roles for this have been proposed, including ribosome preservation and preparation of cells for the reinitiation of growth upon reinoculation into rich medium (6, 25, 31, 35). In E. coli, the dimerization of two 70S ribosomes requires the participation of RMF and HPF, while the binding of YfiA leads to inhibition of translation (6). While it has long been known that RMF is necessary for E. coli to create 100S ribosome particles (1, 3), these 100S particles were traditionally thought to provide a means for the sequestration of ribosomes and to slow down translation by reducing the number of active 70S ribosomes (4). Others have proposed that RMF is involved in many stress responses (9–11), one being to increase ribosome stability (3, 27). Ribosomes are also thought to be degraded during stationary phase as a source of nutrients, as reflected by acridine orange staining. Here, we sought to investigate the long-term effects of the absence of RMF and to confirm that, during both stationary phase and long-term stationary phase, RMF acts as a barrier against the excess degradation of ribosomes through its participation in generating 100S particles.

Our data show that despite lacking *rmf*, the mutant could grow and survive in monoculture similarly to wild-type cells under laboratory conditions for at least 12 days (Fig. 1A). This contrasts with the observation made by Yamagishi et al. (4), who showed that *rmf* mutant cells lost viability after 5 days in stationary phase. This difference may be explained by several factors. First, previous studies have shown that different media can confer different survival phenotypes in long-term batch cultures (21). While our experiments were performed in LB medium, Yamagishi et al. (4) used E medium, a rich defined medium that includes Bacto peptone, Casamino Acids, peptone, and glucose and is buffered. Furthermore, 0.4% glucose, the concentration used in those experiments, has been shown to reduce the long-term viability of E. coli when grown in batch culture, due to increased levels of glycation and other products of reactive oxygen species (36). More similar to our observation, Bubunenko et al. (12) showed that *rmf* did not reduce the viability of cells after 8 days in batch culture when cultured in LB medium. Along with the data presented here, it appears that *rmf* is not essential for the long-term viability of cells when grown under monoculture conditions. One hypothesis that could explain the nonessentiality of RMF is that in the post-death-phase environment, cells encounter various amounts of detrital nutrients that may be scavenged. The availability of novel nutrients in this post-death-phase environment allows more-fit cells, including *rmf* mutant cells, to resume growth and divide. The availability of novel nutrients that are released from the detritus makes it possible for 70S ribosomes to resume activity, thus relaxing the need for RMF during long-term stationary phase. In wild-type cells, we hypothesize that the 100S particles dissociate back into 70S ribosomes during long-term stationary phase. This is consistent with the observation that long-term stationary-phase cells stained with acridine orange dye appear orange in color (D. Siegele and R. Kolter, personal communication). Under coculture conditions, however, different phenotypes were observed (Fig. 1B). The first was the phenomenon we refer to as the “dip,” identified as a significant loss of cell viability of the *rmf* mutant followed by a partial recovery in cell counts over time. Though the *rmf* mutant was able to regain some cell density after the dip, growing ∼10-fold over 3 days, the mutant-cell yield was ultimately ∼100-fold lower than that of the wild type. Here, our data differ from the data of Bubunenko et al. (12), since we did not observe the same degree of death reported in their competition system. This may be explained by the differences in initial culture conditions; they used a coculture system initiated by mixing two stationary-phase cultures 50:50 (vol/vol), which competed the two strains from an already high density. Here, however, the two strains were cocultured from a low initial cell density, which allowed outgrowth through the stationary phase. This outgrowth could affect nutrient availability in the environment throughout the lag, log, and stationary phases, which might affect the death phase significantly.

In competition with wild-type cells, *rmf* mutant cells demonstrated a second lag phase from minute 200 to minute 280 (Fig. 2B). We propose that, due to the lack of RMF, mutant cells have unstable ribosomes that begin to get degraded, causing these mutant cells to pause in order to reestablish conditions favorable for growth. Once these conditions are met, cells reenter growth phase and eventually reach yields similar to those of wild-type cells. This inability to properly regulate entry into stationary phase may relate to the property of 100S ribosomes being translationally inactive. Mutant cells lacking 100S ribosomes may jeopardize their entry into stationary phase by continuing to divide, when instead, macromolecular synthesis, including protein synthesis, should ideally be being adjusted for entry into stationary phase. The outgrowth of the *rmf* strain in both monoculture and competition experiments shows that the mutant is slightly impaired. However, only competition conditions result in the mutant displaying a plateau in growth, indicating that the fitness of the *rmf* mutant is jeopardized by the absence of the RMF protein in the presence of more-fit strains, including the wild type.

Another phenotype that particularly piqued our interest was the difference in overnight cell yields during competition (Fig. 1B). Though relatively small compared to the >100-fold difference in yields during long-term stationary phase, this difference was consistent enough that we chose to investigate the phenomenon further. By determining viable cell counts more frequently (every ∼40 min versus once a day), we were able to determine that the *rmf* mutant had a smaller burst of growth than wild-type cells upon entering log phase at minute 120 (Fig. 2A). This poor growth in early log phase was a phenotype that was observed consistently throughout all experiments. This impairment during the transition between the lag and log phases could explain why the cell yields of the *rmf* mutant were consistently lower than those of the wild type. But perhaps more interesting is the fact that the mutant had a significantly higher growth rate than the wild type once they entered log phase, allowing the mutant to reach the same cell yield as the wild type despite exiting lag phase later.

Growth rate has been reported to correlate with the number of ribosomes present in the cell (37). Our results suggest that *rmf* mutant cells may initially detect their lack of ribosomes and overcompensate, ultimately acquiring three times the number of ribosomes as wild-type cells during log phase (Table 1, Fig. 3). This might explain the phenotype of the high growth rate that the *rmf* mutant experienced as cells transitioned into log phase. Typically, wild-type E. coli cells growing in LB medium have an ∼20-min doubling time, as observed here. However, the *rmf* mutant in both monoculture and coculture had much shorter log-phase doubling times, ∼12.8 and ∼13.6 min, respectively. This short doubling time may be explained by the overproduction of ribosomes that the *rmf* mutant cells exhibited during log phase. We posit that during the first 120 min of slow growth that the *rmf* mutant experiences, cells are accumulating the various important monomers (e.g., amino acids and deoxynucleoside triphosphates [dNTPs]) that are necessary for reproduction. Once cell division is initiated, the accumulation of these ribosome components and monomers allows the higher growth rate of the mutant. To investigate this hypothesis of overproduction, we estimated the relative number of ribosomes present within each cell type using the amounts of total 16S and 23S rRNA obtained from the Agilent 2100 Bioanalyzer. This analysis showed that *rmf* mutant cells had more than three times the number of ribosomes as wild-type cells during log phase. Additionally, we found that the ratios of 23S rRNA to 16S rRNA were approximately the same in both strains. Not only does this indicate proper constitution of 70S ribosomes, it also indicates that the increased number of ribosomes in the mutant may have contributed to its increased growth rate. We posit that this oversynthesis of ribosomes was occurring during the extended lag phase and eventually contributed directly to the higher log-phase growth rate (∼13 min) observed (Fig. 2). The opposite was observed in stationary phase; the relative number of ribosomes per cell in the *rmf* mutant was roughly one quarter that of the wild-type cells, likely due to the increased rate of ribosomal degradation in the absence of 100S ribosomes. When these cells were reinoculated into fresh medium, the initiation of growth with a reduced number of ribosomes likely accounted for the later exit from lag phase experienced by the *rmf* mutant. Once conditions for optimal growth were reestablished, mutant cells then entered log phase.

To determine the time required to reestablish optimal growth conditions, we designed an experiment where wild-type and mutant strains were inoculated into fresh cultures from different time points during outgrowth. Upon reinoculation of cells from the end of lag phase, we hypothesized that if the mutant restored its cellular machinery enough to undergo exponential growth, these cells should not have an extended lag phase when reinoculated into fresh, prewarmed medium. By the end of 120 min, the *rmf* mutant did not grow similarly to the wild type in either the monoculture (Fig. 4A) or the coculture (Fig. 5A), suggesting that conditions within the mutant cells were not normal. This result was also surprising because these mutant cells were beginning to enter log phase, where one might suppose that cells should have finished preparing their machinery to replicate. From mid-log phase (150 min) to early stationary phase (320 min), the mutant grew similarly to the wild type in monoculture upon reinoculation (Fig. 4B to D). However, a contrast was observed when comparing the growth in coculture at these same time points (Fig. 5). Here, the presence of the more-fit wild-type competitor seemed to prevent the *rmf* mutant from resuming normal growth, especially for cultures inoculated from minute 320, when mutant cell counts dropped precipitously during late log phase (Fig. 5D). In monoculture, these mutant cells were able to grow similarly to the wild type after being given enough time to replenish their machinery. In competition culture, however, these cells could not compete with wild-type growth no matter how much time was given to allow recovery. This might be explained by the unequal utilization of nutrients between the wild-type strain, which had more ribosomes, and the less-fit *rmf* mutant. Due to the greater degree of ribosomal degradation, it is possible that *rmf* mutant cells were not able to translate at the same rate as wild-type cells. It is also possible that the restoration from degradation was more energy taxing when a cell needed to remake its ribosomal machinery while competing against another strain. This observation relates to the phenotype observed at minute 320 (Fig. 2B), when the mutant entered a “second lag phase,” where growth was halted temporarily. However, despite the growth rate of the mutant being much higher than that of the wild type during the standard outgrowth experiment, that phenotype did not manifest in this reinoculation experiment, perhaps due to the lack of lag phase, when the build-up of materials occurs.

Taken together, our data suggest that the presence of RMF and 100S ribosome particles plays an important role in controlling the growth and the long-term viability of E. coli. In the absence of RMF, the competitive fitness of cells is jeopardized, even though the mutant appears to undergo the five phases of growth comparably to the wild type in monoculture. Cells that do not have RMF overproduce ribosomes during log phase, which could potentially lower their competitive fitness overall. However, the mutant shows a greatly increased doubling rate during log phase, potentially due to the overproduction of ribosomes, which ultimately increases protein production for cell growth. These data show that maintaining the appropriate numbers of functional ribosomes, as well as the ability to transform ribosomes into 100S particles at the proper growth phase, is crucial for the overall fitness of the cell.

## MATERIALS AND METHODS

### Bacterial strains and mutant construction.

All strains used in this study are isogenic and were derived from the W3110 lineage Escherichia coli K-12 strain ZK126 (Δ*lacU169 tna-2*) (38). An isogenic *rmf* null mutant strain (SF2603) was constructed via bacteriophage P1 transduction into ZK126 using a donor strain carrying the *rmf*::Kan allele obtained from the KEIO collection (KEIO strain JW0936) (39). Replacement of the *rmf* gene with the kanamycin resistance cassette was confirmed by PCR (data not shown). ZK1142 is a nalidixic acid-resistant isogenic wild-type strain derived from ZK126 (40, 41).

### Culture conditions, media, and titer assays.

For both monoculture and coculture experiments, strains were incubated in 5 mL LB broth (Lennox) (10 g tryptone, 5 g yeast extract, 5 g NaCl; components from BD) in 18- by 150-mm borosilicate test tubes at 37°C with aeration in a TC-7 roller drum (New Brunswick Scientific). Viable cell counts were measured using the spot titer determination assay (42) with a limit of detection of >1,000 CFU/mL. Cells were plated on LB agar for monoculture experiments and on LB agar with nalidixic acid (20 mg/mL) or kanamycin (50 mg/mL), as appropriate, for coculture experiments.

### Batch culture competition assays.

Direct head-to-head competitions between the parental and *rmf* mutant strains were performed using cells from overnight cultures of both strains coinoculated at 1:1,000 (vol/vol) dilution into 5 mL of LB. Titer determination assays were performed over time to quantify the viable counts of each strain for each time point sampled throughout the stationary phase and long-term stationary phase.

### Lag-phase and log-phase studies.

Both monoculture and coculture experiments were incubated in 5 mL LB broth as described above. To determine viable cell counts throughout lag phase and the transition into log phase, the titers of the cultures were determined at the time of inoculation (time = 0 min) and every subsequent 40 min (through minute 320). For each time point, 10 μL of culture was sampled and the titer determined for viable cell counts. Cells were plated on LB agar for monoculture experiments and on LB agar with the appropriate antibiotic (nalidixic acid or kanamycin) for competition experiments. The calculations of generation times were performed using the equation *G* = *t*/[3.3 × log(*N_t_*/*N*_0_)], where *G* is the generation time, *t* is the time interval between two time points, *N_t_* is the CFU/mL at the second time point, and *N*_0_ is the CFU/mL at the first time point. A linear trendline was fitted to each growth curve for each replicate for both the wild-type and *rmf* mutant strains to obtain the growth rate for each strain, and an *R*^2^ value was generated using Microsoft Excel (*R*^2^ = 0.995 for the wild type in monoculture, 0.999 for the *rmf* mutant in monoculture, 0.995 for the wild type in competition, and 0.985 for the mutant in competition). The growth rates reported are the average values from three experiments for each strain.

### Growth-phase-specific reinoculation experiments.

Monoculture and coculture experiments were performed as described above; however, at the 120-, 150-, 200-, and 320-min time points, 5 μL (1:1,000 dilution) of either ZK1142 or SF2603 was reinoculated into a fresh culture tube of prewarmed 5 mL LB. Then, at the time of reinoculation (time = 0 min) and every 40 min subsequently (through minute 160), 10 μL of culture was sampled to determine viable cell counts as described above. The length of the lag phase was determined as the time from inoculation until the time when the viable cell counts first showed at least a doubling. Paired-sample *t* tests were performed for each time point in every reinoculation experiment for both monoculture and coculture using Microsoft Excel (Fig. 4 and 5).

### Quantitation of 16S and 23S rRNA.

Total cellular RNA was measured using the Agilent 2100 Bioanalyzer (https://www.agilent.com/en/product/automated-electrophoresis/bioanalyzer-systems/bioanalyzer-instrument/2100-bioanalyzer-instrument-228250), following the manufacturer’s instructions. Wild-type and *rmf* mutant strains were incubated overnight as described above. Whole-cell RNA was purified using the Qiagen RNeasy minikit, following the manufacturer’s instructions, although the volume of reagents used was doubled to improve yield. After eluting the samples into 100 μL of elution buffer, a Nanodrop spectrophotometer was utilized to measure both concentration and purity. Samples were then diluted to obtain 1 to 3 ng/μL of purified RNA, and 1 μL was loaded onto the Bioanalyzer chip. Culture samples were taken 150 min after reinoculation for log-phase samples and 24 h after reinoculation for stationary-phase samples. We calculated the 16S and 23S rRNA masses by multiplying the area percentage of each rRNA type by the total RNA in picograms. The area percentages were obtained from the 16S and 23S peaks of the Bioanalyzer graphical output (43) by subtracting the signal of the ladder control lane from the signal of each of the experimental lanes. We then calculated the number of ribosomes based on 16S or 23S quantities using the formula *R* = (*C* × *f*/*d*)/*W*, where *R* is the number of ribosomes, *C* is the concentration of the total rRNA obtained from the Bioanalyzer and corrected for its dilution factor, *f* is the percentage of 16S or 23S from the total RNA, *d* is the total number of cells, and *W* is the weight of 16S or 23S rRNA in picograms, obtained by converting the weight of rRNA from Daltons to picograms (https://www.idtdna.com/pages/education/biotech-basics).
